# A commentary on studies of brain iron accumulation during ageing

**DOI:** 10.1007/s00775-024-02060-2

**Published:** 2024-05-12

**Authors:** Mark J. Hackett

**Affiliations:** 1 School of Molecular and Life Sciences, Curtin University, Perth, WA 6845 Australia; 2 Curtin Health Innovation Research Institute, Curtin University, Perth, WA 6102 Australia

**Keywords:** Metallomics, Senescence, X-ray fluorescence, XFM, Brain rust

## Abstract

Brain iron content is widely reported to increase during “ageing”, across multiple species from nematodes, rodents (mice and rats) and humans. Given the redox-active properties of iron, there has been a large research focus on iron-mediated oxidative stress as a contributor to tissue damage during natural ageing, and also as a risk factor for neurodegenerative disease. Surprisingly, however, the majority of published studies have not investigated brain iron homeostasis during the biological time period of senescence, and thus knowledge of how brain homeostasis changes during this critical stage of life largely remains unknown. This commentary examines the literature published on the topic of brain iron homeostasis during ageing, providing a critique on limitations of currently used experimental designs. The commentary also aims to highlight that although much research attention has been given to iron accumulation or iron overload as a pathological feature of ageing, there is evidence to support functional iron deficiency may exist, and this should not be overlooked in studies of ageing or neurodegenerative disease.

**Graphical abstract:**

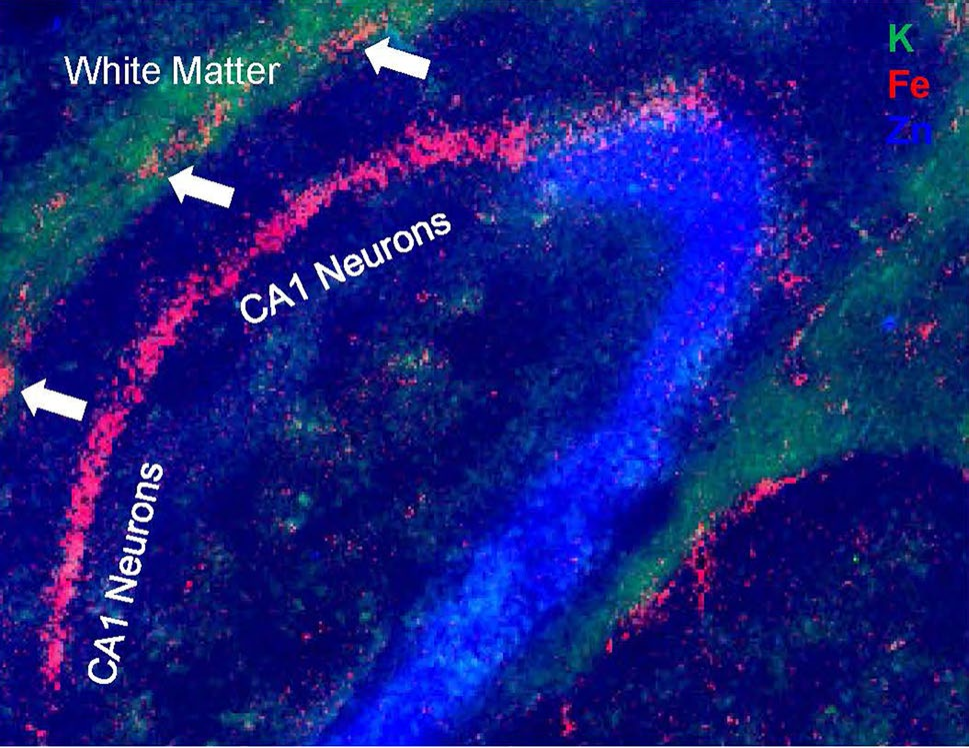

Changes to brain metal homeostasis during natural ageing and neurodegenerative disease have been under investigation for several decades, in particular age-related increases in brain iron (Fe) content. Due to the propensity of Fe to catalyse free radical generation through classic Fenton-like chemistry, many researchers logically propose a pathological link between age-related increases in brain Fe and heightened oxidative stress [1–13]. Cognitive decline and increased risk of neurodegenerative disease are the proposed outcomes of age-related Fe increase [5–7, 14], with some suggesting metal chelation therapy as a possible therapeutic strategy [11, 15–20].

Despite the widely supported view that brain Fe increases during ageing, this article aims to provide a critical commentary of the literature on this topic. First, this paper will begin with a brief review of the role of Fe in healthy brain function and the analytical tools available to study brain Fe. Second, this article will highlight that the published literature does not adequately differentiate between “adulthood” and “senescence” (the biological condition of deterioration with age) when studying Fe homeostasis in the ageing brain. The majority of studies of “ageing” in fact only investigate adulthood but not senescence. Given that age is the greatest risk factor for neurodegenerative disease [21, 22], and the fact that the ageing process itself results in cognitive decline during senescence [22, 23], it is important to differentiate between how brain Fe content (as well as distribution and speciation of Fe) changes during both adulthood and senescence. The article aims to summarise what is currently known about Fe changes in the brain during adulthood and senescence based on the published literature. Lastly, while much of the literature studying brain Fe during ageing has focussed on Fe accumulation, there may also be a role for functional Fe deficiency [24–27]. This commentary will, therefore, conclude by highlighting the concept of functional Fe deficiency during ageing and senescence, and how despite elevated total brain Fe content, chemical pathways and physiological processes responsible for cognitive decline may be driven by localised Fe deficiency.

## What is the role of Fe in the brain and how do we detect it?

**Fig. 1:**
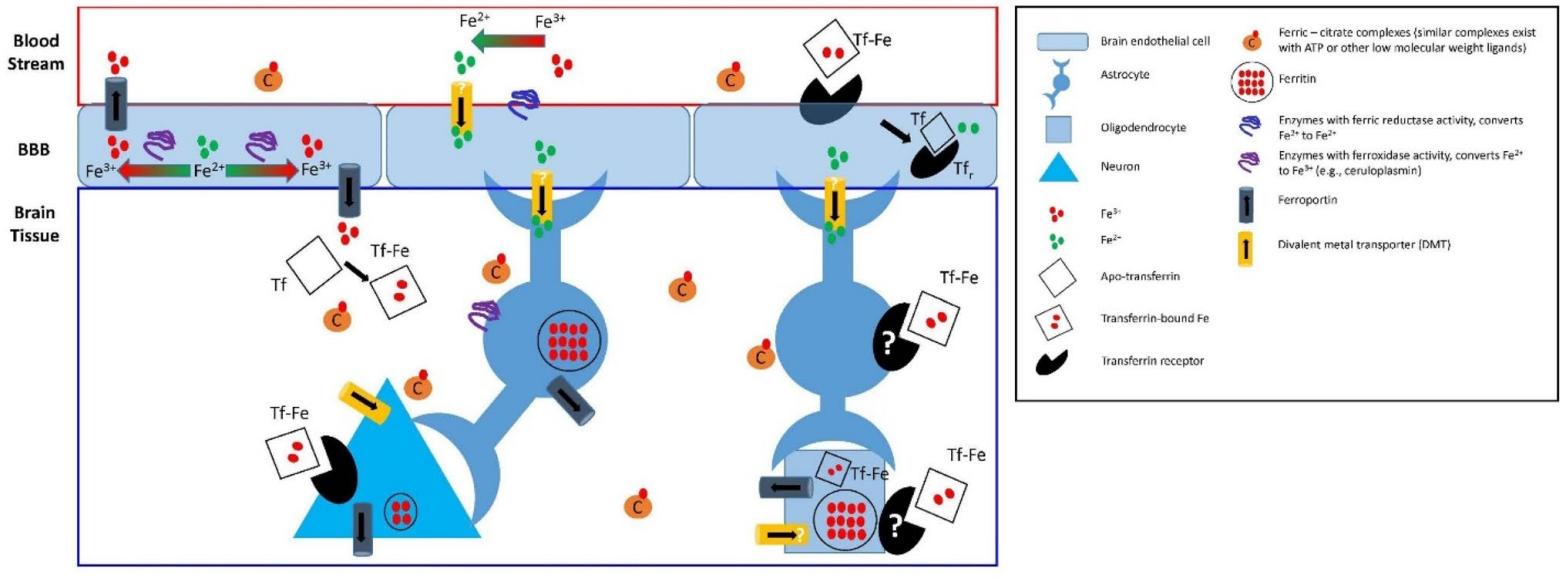
Schematic of Fe transport into and within the brain: A large fraction of Fe destined for the brain is transported in the blood stream bound to transferrin (the main Fe-transport protein), however substantial pool of non-transferrin bound Fe is also present, such as Fe bound to albumin or in low-molecular-weight complexes with molecules such as ATP or citrate. At the blood–brain barrier (e.g. endothelial cell apical surface), transferrin bound Fe enters endothelial cells via transferrin receptor-mediated internalisation on the luminal surface. Alternatively, labile of Fe^3+^ may be reduced by reductases on endothelial cell surface, which could enable Fe^2+^ via divalent metal transporters (DMT). Direct entry of low-molecular-weight complexes of Fe^3+^ into endothelial cells, such as Fe^3+^–citrate complexes could also be possible. Within cell cytoplasm, Fe^2+^ may be oxidised via enzymes with ferroxidase activity, enabling subsequent export of Fe^3+^ through ferroportin. Release of Fe^3+^ from abluminal membrane of endothelial cells, via ferroportin, is one pathway through which Fe^3+^ could enter the brain interstitial fluid. Once in interstitial fluid, Fe^3+^ may exist as low-molecular-weight or labile Fe^3+^ complexes, it may be taken up via apo-transferrin, or it may be reduced to Fe^2+^ where it can enter other brain cells (astrocytes, oligodendrocytes, neurons, etc.) through membrane-bound DMT. Transferrin receptors on cell membranes of neurons present a major pathway for Fe entry, from the interstitial Fe pool, into brain cells. It is not clear if transferrin receptors enable Fe entry into glia in vivo. Similarly, there is conflicting literature regarding the in vivo role of DMT between different brain cells. Fe^3+^ bound to low-molecular-weight complexes, such as Fe^3+^–citrate represents another pathway through which Fe enters brain cells from the interstitial fluid. Excess Fe within brain cells is sequestered/stored as ferritin, with glial cells displaying far greater capacity for Fe storage than neurons. Note: This schematic is for general illustration purposes, and is not meant to convey an exhaustive summary of Fe import and transport mechanisms. White arrows indicate pathways where diverging opinions exist in the literature. Schematic adapted from literature references [25, 34–37]

Although unregulated Fe accumulation in cells is harmful, Fe is essential for healthy function of the cell. Ubiquitous roles for Fe needed in all cells include: incorporation into ferrodoxins of the electron transfer chain [28]. Fe inclusion in cytochromes (ATP production) [29] and acotinase in the citric acid cycle [30], and modulating signalling pathways through binding to RNA (iron regulatory elements, or IREs) [31]. Unique to brain cells, Fe serves as a co-factor in enzymes required for neurotransmitter synthesis (phenylalanine hydroxylase, tyrosine hydroxylase, tryptophan hydroxylase) [25] and lipid synthesis by oligodendrocytes for myelination of axons [32, 33]. A generalised schematic (adapted from the literature) showing Fe transport into the brain and between brain cells, which is needed to meet the above function requirements of brain cells, is provided in Fig. 1 [25, 34–37].

Cellular Fe storage is clearly critical to enable ready availability of Fe to facilitate the above-mentioned processes, which is achieved by ferritin. A detailed discussion of ferritin is beyond scope of this article, but most if not all readers will be aware of its importance as a key Fe storage and Fe regulatory protein [38–43]. The successful storage of intra-cellular Fe in ferritin, when not in use, is critical to prevent catastrophic oxidative damage that would otherwise occur to cells in an unregulated Fe rich environment [43].

Despite the clear necessity of Fe for brain cells and in particular neurons (for neurotransmitter synthesis and associated metabolic support), it seems unexpected that many studies using classical Fe histochemistry (Perls Prussian Blue stain) fail to detect Fe in neurons [44–48]. To quote the literature (*Sands et al.*,) “Histochemical methods of detecting iron in the rodent brain result mainly in the labelling of oligodendrocytes, but as all cells utilise iron, this observation suggests that much of the iron in the central nervous system goes undetected” [44]. The absence of Perl’s (or Turnbull’s) staining in neurons is misleading, as failure to produce Perl’s positive staining does not indicate an absence of Fe, but rather an absence of histochemically detectable Fe. Ourselves and others have now shown, using direct spectroscopic measurement (X-ray fluorescence microscopy or proton-induced X-ray emission spectrometry), that although neurons contain less Fe than glial cells, they do indeed contain a substantial Fe pool (~ 0.5–1 mM) [49–53]. The discrepancy between Perl’s histochemistry and direct spectroscopic measurement could be due to the specific chemical forms of Fe found in neurons. Specifically, neurons likely contain substantial amounts of low-molecular-weight or labile Fe, which is in agreement with their requirements of Fe as cofactors for neurotransmitter synthesis. Indeed, more recent modification to the Perl’s method, with protocol alterations aimed at minimising loss of labile Fe, do indeed show staining of neuronal cytoplasm [44, 54]. The discrepancies regarding measurement of Fe concentration and distribution described above demonstrate some of the challenges when studying brain Fe, and highlight that careful consideration should be given to the analytical measurement used. In addition, due to the heterogeneous distribution of Fe in the brain (described in detail below), consideration needs to be given to the specific sample being analysed, such as whole tissue homogenate, micro-dissected tissue region, or thin tissue sections.

**Fig. 2:**
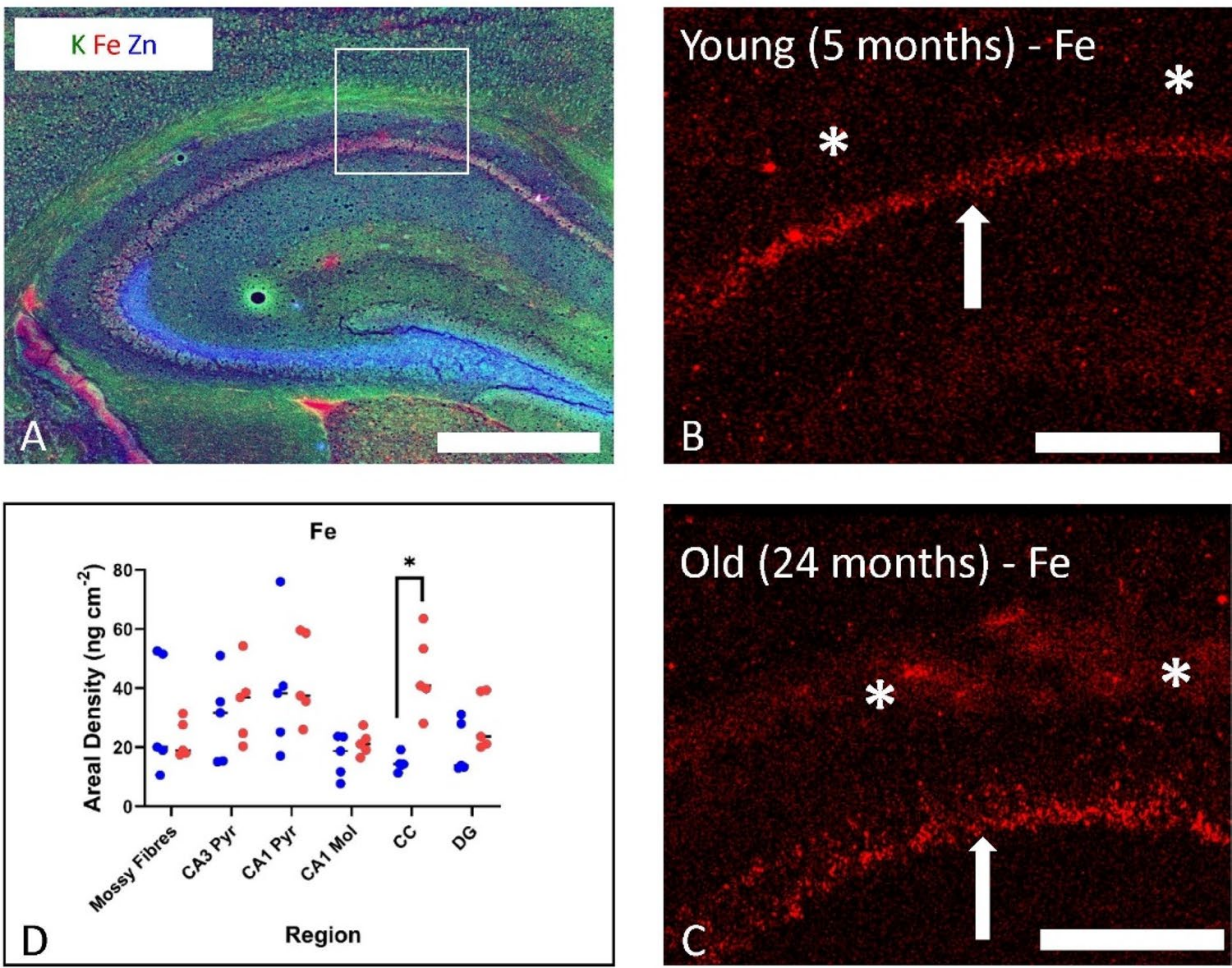
Direct elemental mapping techniques such as X-ray fluorescence microscopy (XFM) have emerged as valuable tools to characterise brain Fe homeostasis during ageing. **A** Shows a multi-colour XFM overlap image of K (green), Fe (red) and Zn (blue) in a mouse hippocampus. White box in **A** shows approximate anatomical location of the elemental maps shown in panels **B** and **C**. **B, C** XFM elemental maps of Fe in a 5-month-old and 24-month-old C57Bl6 mouse. White arrows indicate location of hippocampal CA1 pyramidal layer where age-related Fe increase was not observed, while asterisks highlight the corpus callosum white matter where age-related Fe increase is observed, as described in **D** statistical analysis. Scale bar in A = 500 µm, B, C = 200 µm. Panel A reproduced with permission from reference [57], and Panels **B**–**D** reproduced with permission from reference [45]

While Perl’s histochemical methods clearly have limitations for detection of labile Fe and indeed the total Fe pool, it is a robust and easily accessible technique that enables study of Fe deposition and Fe storage, and the method has revealed a wealth of important knowledge of changes in Fe deposition in the ageing brain [40–42, 49, 55]. To complement Perl’s staining, many researchers now use direct elemental mapping techniques such X-ray fluorescence microscopy (XFM) [49, 56–61]. proton-induced X-ray emission spectrometry (PIXE) [51–53]. secondary ion mass spectrometry (SIMS) [62, 63]. or inductively coupled plasma mass spectrometry (ICP-MS) [8, 13]. These techniques are well documented in the literature, and they provide mapping capabilities at cellular resolution (e.g. spatial resolution on the order of microns) or sub-cellular resolution (e.g. sub-micron spatial resolution). Representative examples of XFM elemental mapping to study brain Fe distribution are shown in Fig. 2. It should be noted that the above techniques typically do not reveal information on chemical speciation of Fe; however, this can be obtained from a variant of XFM, micro-X-ray absorption near-edge spectroscopy (micro-XANES) [64–66], or Mössbauer spectroscopy [67].

A limitation of the above-mentioned elemental mapping techniques is that they are mostly suited to analysis of tissue sections ex vivo. Care must be taken to ensure sample preparation does not change the distribution or chemical form of metal ions from the in vivo state [68–70]. Unfortunately, in vivo assessment of Fe is limited, and techniques capable of studying Fe levels and Fe speciation in vivo, such as MRI [71, 72], do not offer the same level of spatial resolution as the elemental mapping techniques described in the previous paragraph. Overall, no single analytical tool can provide a complete picture of brain Fe homeostasis, and the above-mentioned tools should be complemented with other analytical methods such as immuno-fluorescence, western blot, RNA sequencing (and more) wherever possible.

### Where is brain Fe located?

While the brain is Fe rich, the distribution of Fe is highly heterogeneous between different brain regions and different cell types, with large variation also observed at the sub-cellular level. It is well established that Fe concentration varies between specific brain regions, such as basal ganglia, hippocampus, cortex, cerebellum, pons and other structures [11, 73]. Of particular interest to this commentary is hippocampal Fe concentration, as the hippocampus is a brain region critical to memory function, and it is often the first brain region impacted during cognitive decline associated with ageing or dementia [74, 75]. Owing to its implication in memory loss, metal distributions in the hippocampus have been investigated in detail, by multiple research groups, and the hippocampus provides an excellent example of the cellular and sub-cellular variation in Fe content that can occurs within the brain. Fe distribution within the hippocampus although heterogeneous, displays a highly consistent pattern of distribution [49, 50, 58, 60, 76, 77]. Specifically, multiple X-ray fluorescence microscopy studies reveal that the neuronal layers of the hippocampus, such as the pyramidal neurons of cornu Ammonis layers 1, 2, 3 (CA1, CA2, CA3) and granule cell layer of the dentate gyrus contain more Fe than surrounding molecular layers, stratum oriens or stratum radiatum layers (dentate gyrus molecular layer is the most Fe enriched of the molecular and stratum layers) [76]. Within the CA layers, the CA1 layer contains appreciably more Fe than the CA2 or CA3 layers [50]. Although the neuronal layers of the hippocampus contain the greatest amount of Fe relative to the other hippocampus tissue layers, the neurons are not the most Fe-enriched cell of the hippocampus. Glia and microglia, contain appreciably more Fe than neurons, with Fe content reported to be greatest in oligodendrocytes, followed by microglia, and then astrocytes [52, 53, 78]. In all cells, neurons, microglia and glia, Fe distribution appears highly localised, with Fe-enriched punctate deposits observed both in situ within tissue sections [51–53, 79], and also *i*n vitro from cell grown in culture [80–83]. The exact organelles responsible for the punctate sub-cellular Fe distribution and the chemical form of the Fe in these deposits still remain to be elucidated.

## What is meant by “ageing”, “aged” and “senescence”?

In the broadest definition, studying changes in brain Fe content during “ageing” could mean measurement of brain Fe across any two (or more) time-points across the lifespan. However, context is important, and the vast majority of studies of “brain Fe during ageing” aim to investigate mechanisms of cognitive decline or neurodegenerative diseases, which are processes/diseases associated with senescence.

**Fig. 3:**
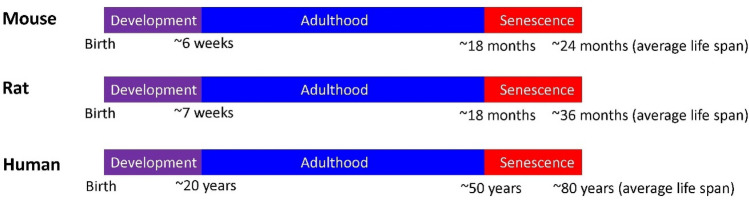
Schematic showing the approximate association between age and biological timeline of life (development, adulthood, senescence) in mice, rats and humans. The majority of pre-clinical studies in rodents have characterised changes in brain Fe levels during adulthood (blue shaded region), with very few studies characterising brain Fe homeostasis across the period of senescence (red shaded region). Schematic developed from data contained in references [69] and [70]

In the context of studying brain Fe during ageing, it is important to differentiate between “adulthood” and “senescence”. In rodent models, adulthood can be difficult to define, so often sexual maturity at adolescence is used to delineate, and therefore “adulthood” regarded as the period from sexual maturity to cessation of ability to sexually reproduce. Puberty is generally regarded to start at ~ 6 weeks for mice and ~ 7 weeks in rats [84, 85]. In humans, adulthood is often described as closure of the growth plate, which occurs at ~ 20 years of age [84, 85]. Similar to adulthood, providing a precise definition of senescence is also difficult between rodents and humans, but often cessation of reproduction function is used as a marker (using onset of menopause in women as a human time-point), which equates to ~ 18 months in mice, and ~ 50 years in humans [84, 85]. Although direct translation of rodent age to human age is not possible, and there are gender differences, the approximate time-points of “adulthood” (i.e. the time from the start of adulthood until onset of senescence) spans 6 weeks–18 months in mice, 7 weeks–18 months in rats and approximately 12–50 years in humans [84, 85]. The period of senescence is, therefore, described as ages greater 18 months in mice and rats, and greater than ~ 50 years in humans [84, 85]. A schematic of the above biological timelines for mice, rats and humans is presented in Fig. 3.

It is important to note that there are a number of well-defined physiological and neurochemical differences between the adult and senescent brain, particularly with respect to myelination, dendritic plasticity and neurogenesis [86]. Myelination, which is an energy-consuming and Fe-demanding process, is not complete at the start of adulthood, and in fact the myelin content of the rodent and human brain continues to increase until mid-late adulthood, but decreases in senescence [87, 88]. The greatest period of synaptic plasticity and ability to form new dendrites is found in the developing brain, but the adult brain still displays more plasticity than the senescent brain [89]. Further, there is a remarkable drop in neurogenesis [90], rates of neuronal protein synthesis [91], and synaptic plasticity[89] in the senescent brain relative to the adult brain. Lastly, the senescent brain displays a heightened state of inflammation relative to the adult brain [92].

Unfortunately, many of the studies that have investigated brain Fe during ageing do not actually study the period of senescence, i.e. mice or rats older than 18 months of age are not used. In studies that do use animals with ages corresponding to senescence, many have incorporated an experimental design that consists of only two time-points (generally one in early-mid-adulthood and one at the beginning of senescence). Although a two time-point design is sufficient to demonstrate that the senescent brain contains greater Fe content relative to a period earlier in life, it is not possible to determine if Fe accumulation occurs during adulthood or senescence (or both) when only using two time-points. To differentiate between changes in brain Fe levels throughout adulthood and senescence, a minimum of 3 time-points is required (e.g. one in adulthood, one at the interface of late adulthood and early senescence, and one in senescence).

## Is elevated Fe in the senescent brain the result of Fe accumulation during senescence or Fe accumulation during adulthood?

There is an abundance of published literature demonstrating that the brains of senescent animals contain more Fe than the brains of animals earlier in life. Of studies that only examined two age points, Sato et al. demonstrate elevated brain Fe in senescent 22-month-old C57BL/6 mice relative to early adulthood mice (4 months old) [9]. Ellison et al. (see Fig. 2B–D) demonstrated elevated Fe in the white matter of 24-month-old C57Bl/6 mice, relative to 5-month-old animals [49]. Focht et al., demonstrated elevated brain Fe in 24-month-old Fischer rats relative to 4-month-old animals [55], and although the study of Liu et al., did not extend to senescence, they demonstrate elevated brain Fe in 64 week-old (~ 15 month) C57Bl/6 mice relative to 8-week-old (~ 2 month) mice.

While the above-cited studies provide clear evidence that the senescent brain contains greater Fe content that the young adult brain (in rodents), they do not identify when the Fe accumulation has occurred. Studies that have however, examined brain Fe content at multiple time-points across adulthood and senescence do not support increasing brain Fe content during ageing (in rodents or humans). Rather, the published literature indicates brain Fe accumulates during adulthood with the increasing content appearing to cease at the onset of senescence. For example, the works of Massie et al. quantified brain Fe in male C57BL-6 J mice from 37 to 888 days, which revealed substantial increases in brain Fe concentration in the first year of life, but no significant increase after [3]. In another study, Morita et al. quantify brain Fe in female B10BR mice aged 2, 6 and 10 months old (i.e. a timeline that spans adulthood, but does not include senescence which starts at 18 months in mice), which revealed Fe levels in 10 month-old mice were increased relative to 2 month-old mice, but there was no difference in brain Fe levels between 6 and 10 month-old animals. This suggests that Fe levels have increased during the earlier part of adulthood (e.g. 2–6 months), but not later in adulthood (6–10 months). Takahashi et al. studied brain Fe content in C3H mice and Wistar rats across ages from 1 to 104 weeks (1 week–24 months). Their results showed increases in Fe over the time span of 1–17 weeks, but again no increase was observed from 17 to 104 weeks. The studies by Belaidi et al. analysed brain Fe content across adulthood and senescence, studying brain Fe in C57BL6/SV129 mice aged 8,12, 18 and 22 months [13]. While Fe levels were elevated in mice aged 22 months relative to 8 month mice, there was no increase in brain Fe content from 18 to 22 months (i.e. brain Fe content did not increase during senescence) [13]. The works of Yoo et al. assessed relative hippocampal Fe content in Gerbils aged 1, 3, 6, 12, 18 and 24 months, using Perls histochemical staining of tissue sections, [93] and similar to the studies above, although the Fe content of the senescent hippocampus (18 and 24 months old) was increased relative to earlier time-points in life, there was no increase in histochemically detected Fe between 18 and 24 months [93]. Taken together, the literature presented above robustly demonstrates that the period of brain Fe accumulation appears to be during adulthood (often early to mid-adulthood) and not during senescence.

Similar to rodent models, the senescent human brain has also been reported to display elevated Fe content relative to earlier time-points in life. As with rodents though, the time period of brain Fe accumulation appears to be adulthood, not senescence: Zecca et al. studied Fe content of the substantia nigra in men from birth to 90 years old, revealing robust increase in brain Fe earlier in life, but not increasing during late adulthood or senescence. [94] Similarly, Markebery et al. also reported significant Fe accumulation earlier in life but not during late adulthood or senescence in humans [95]. Taken together, the results from animal and human studies appear to partially contradict the frequently published statement “brain Fe accumulation during ageing”. The Fe content of the aged brain is clearly higher when compared to earlier time-points in life, but this increase in Fe content appears to be driven by Fe accumulation during adulthood (often early adulthood), and the published literature does not support brain Fe accumulation during late adulthood or during senescence. This raises important questions about how Fe transport and handling is changing during late adulthood and senescence. Further, it is interesting that there an association between ageing and oxidative stress in the brain, but brain Fe is not increasing during late adulthood and senescence. One possible explanation is that the total content of brain Fe is not the best predictor of oxidative stress, but rather the key driver is the specific chemical form of Fe, and the cell/organisms ability to handle that Fe. This is supported by recent studies in C. elegans, which do indeed suggest that total Fe content is not the key determining factor of age-related pathology, but rather the organisms ability to handle specific forms of Fe is more critical [65].

## Brain Fe is critical for cognition and synaptic plasticity, and increases rapidly during neurodevelopment

Before presenting the concept of functional Fe deficiency during ageing, for context, it is worthwhile to summarise how brain Fe content changes early in life (neurodevelopment). The Fe content of the brain is generally low following birth, and rapidly increases after [38, 39, 60, 96]. In mice and rats, the period of rapid brain Fe accumulation appears to extend to at least 2–3 months of age [38, 60, 97], and appears to coincide with a permeable or incompletely formed blood–brain barrier [38, 39, 96]. Of note, Fe deficiency during this critical stage of brain development can have lasting effects on cognitive function [96]. Interestingly, many of the symptoms that follow Fe deficiency during neurodevelopment mirror those seen in the senescent brain, and also during neurodegenerative disease. Pathology that is common between Fe deficiency in neurodevelopment and ageing/neurodegenerative disease include: reduced levels of PSD-95 (a key protein required for dendrite plasticity) [96, 98], reduced levels of MAP-2 (a marker of dendrite density) [99], reduced cytochrome-c oxidase activity [100, 101], reduced mitochondrial respiration [102], impaired dopamine synthesis [101, 103–105], and hypomyelination [101, 105]. A simple comparison of the commonalities between Fe deficiency during brain development and ageing or neurodegenerative disease suggests a role of Fe deficiency during senescence and brain disease is plausible.

## Functional Fe deficiency during ageing?

There is growing evidence that supports a role of functional Fe deficiency as a contributing factor to brain ageing and neurodegenerative disease. The theory has recently been stated in the literature as the “Azalea hypothesis” [27], and essentially postulates that excessive Fe sequestration during ageing or neurodegenerative disease creates conditions of functional Fe deficiency in neurons, disrupting normal/healthy neuron function. It should be noted that this theory is not completely new, with others having previously proposed that localised or functional Fe deficiency could occur during ageing or neurodegenerative disease [24, 106–108]. In support of this theory are recent transcriptomic analysis that indicate a degree of overlap in gene expression between brain tissue in the early stages of Alzheimer’s disease pathology and genes associated with anaemia [26]. Other studies have used genetic manipulation of mice to create functional Fe deficiency, through ablation of Iron Regulatory Protein genes, which post-transcriptionally causes mis-regulation of proteins involved in Fe transport (transferrin) and Fe storage (ferritin) [102]. The impact from genetic manipulation of iron regulatory protein genes are motor neuron loss, which is attenuated if capacity for Fe sequestration is reduced, which provides a further link between Fe sequestration and functional Fe deficiency [102]. Inflammation has been proposed as a key pathological driver of Fe sequestration [106], and it is well established that expression of key Fe regulatory proteins DMT1, FPN1, and hepcidin are altered in response to inflammatory cytokines, promoting Fe sequestration [109, 110]. Specifically, in response to pro-inflammatory cytokines such as tumour necrosis factor-α (TNF-α) and interleukins: DMT1 is upregulated in brain cells (increasing capacity for Fe accumulation), hepcidin is upregulated and FPN1 is downregulated (decreasing capacity for Fe efflux and/or transport), and ferritin expression is increased (increasing capacity for Fe storage [109–112]. Therefore, although relatively underexplored, it is possible that heightened inflammation during brain ageing drives a shift in Fe homeostasis to favour Fe sequestration, and in doing so creates localised functional Fe deficiency. Lastly, given that Fe homeostasis is often related to homeostasis of other transition metals such as Cu, and Cu is also known to accumulate in glial cells in the ageing brain [113], investigation of common physiological pathways that promote both Cu and Fe accumulation may of interest for future work.

### Conclusion and future outlook

Based on the published literature studying brain Fe content, the frequently used statement “brain Fe increases during ageing” has the potential to be misleading depending on how “ageing” is understood or defined, and unfortunately in many published studies it is left undefined. As has been presented in this article, most studies have not studied brain Fe during the period of both adulthood and senescence and in the few studies that have, brain Fe content has been shown to increase during adulthood but not senescence. As the period of senescence is of critical importance to cognitive decline during natural ageing and also increased risk of neurodegenerative disease, continued use of the expression “increased brain Fe during ageing” (or similar expressions) is misleading and not supported by the current published literature. Future studies should be encouraged to specifically designate which biological period of life age-related changes in Fe (or other metals) occur (e.g. development, puberty, adulthood, senescence). It is hoped that this article has highlighted the need for greater characterisation of brain Fe homeostasis during the period of senescence, with a focus not only on amount and distribution, but also on chemical form (speciation) of Fe. Lastly, although much research attention has been given to the role that elevated Fe may have in driving oxidative stress, it is hoped that future studies will pay greater attention to explore the potential roles of functional Fe deficiency during brain ageing.

## Data Availability

As this is a review/commentary, there is no applicable data availability statement.
